# Application of multi-trait Bayesian decision theory for parental genomic selection

**DOI:** 10.1093/g3journal/jkab012

**Published:** 2021-01-20

**Authors:** Bartolo de Jesús Villar-Hernández, Sergio Pérez-Elizalde, Johannes W R Martini, Fernando Toledo, P Perez-Rodriguez, Margaret Krause, Irma Delia García-Calvillo, Giovanny Covarrubias-Pazaran, José Crossa

**Affiliations:** 1 Colegio de Postgraduados, Montecillos, Edo. de Mexico, CP 56264, Mexico; 2 Universidad Autonoma de Coahuila, Saltillo, CP 25280, Mexico; 3 International Maize and Wheat Improvement Center (CIMMYT). Km 45 Carretera México-Veracruz, El Batán Km. 45, CP 56237 México

**Keywords:** loss function, multi-trait selection, parental selection, genomic selection, wheat multi-trait data, genomic prediction

## Abstract

In all breeding programs, the decision about which individuals to select and intermate to form the next selection cycle is crucial. The improvement of genetic stocks requires considering multiple traits simultaneously, given that economic value and net genetic merits depend on many traits; therefore, with the advance of computational and statistical tools and genomic selection (GS), researchers are focusing on multi-trait selection. Selection of the best individuals is difficult, especially in traits that are antagonistically correlated, where improvement in one trait might imply a reduction in other(s). There are approaches that facilitate multi-trait selection, and recently a Bayesian decision theory (BDT) has been proposed. Parental selection using BDT has the potential to be effective in multi-trait selection given that it summarizes all relevant quantitative genetic concepts such as heritability, response to selection and the structure of dependence between traits (correlation). In this study, we applied BDT to provide a treatment for the complexity of multi-trait parental selection using three multivariate loss functions (LF), Kullback–Leibler (KL), Energy Score, and Multivariate Asymmetric Loss (MALF), to select the best-performing parents for the next breeding cycle in two extensive real wheat data sets. Results show that the high ranking lines in genomic estimated breeding value (GEBV) for certain traits did not always have low values for the posterior expected loss (PEL). For both data sets, the KL LF gave similar importance to all traits including grain yield. In contrast, the Energy Score and MALF gave a better performance in three of four traits that were different than grain yield. The BDT approach should help breeders to decide based not only on the GEBV per se of the parent to be selected, but also on the level of uncertainty according to the Bayesian paradigm.

## Introduction

Genetic improvement of plants and animals is based on selecting and intermating the best performing parents to form the next improved population. The selection of which candidates to advance to the next breeding cycle is based on the breeding value (BV) and net genetic merits of multiple traits, and decisions are made based on their performance in crop fields and greenhouses. In genomic selection (GS), the genomic estimated breeding value (GEBV) of unobserved candidates is predicted using only genotypic information and a statistical model trained with phenotypic and genotypic data of individuals in the training population (Meuwissen *et al.*, 2001).

The main decision in breeding is how to select the parents that maximize the response to selection (*R*) expressed as the difference between the mean of the offspring of the selected individuals ($\mu_{2}$), and the mean of the original population ($\mu_{1}$) ($R=\mu_{2}-\mu_{1}$). This decision can be achieved by different methods. The conventional strategy for selecting the best parents in a single trait is selection by truncation, which consists of ranking in descending or ascending order based on GEBV (depending on the desired direction of selection) and selecting some fraction of the top lines. With current genomic information, the optimum contribution theory (OCT) is a method for selecting the parental candidates with the aim of increasing the genetic gain by optimizing the genetic contribution of each individual to the next generation for a given rate of inbreeding (Henryon *et al.* 2014; Woolliams *et al.* 2015). Under the OCT method, the selection of candidates is based on high genetic merit, and on the relationship among the candidates for selection. In general, the main question is how to balance high genetic gains in the next generation while maintaining genetic diversity (Kinghorn 2011; Cowling *et al.* 2019). Akdemir and Sánchez (2016) optimized genomic mating between parents under GS by applying a method that uses a function that combines measures of inbreeding as part of the objective function being minimized for a single trait. Furthermore, Han *et al.* (2017) selected the donor parents for the introgression of alleles to recipient individuals by proposing an optimized algorithm. Bulmer (1980) and Gianola and Fernando (1986) showed that, for a single trait, the conditional expectation of each candidate for selection maximizes the mean of true genotypic values and minimizes a squared loss function.

Decisions made during GS are based solely on the GEBV of the candidates for selection. Genomic approaches (1) estimate the GEBV through a statistical model and information about the unobserved (genotyped) individuals (candidate population) using the phenotypic and genotypic data of their parents, (2) rank the lines based on GEBV, and (3) select the top-ranking lines. Recently, Villar-Hernández *et al.* (2018) proposed a method based on Bayesian decision theory (BDT) for selecting the best candidates (in a single trait or in multi-trait) that maximize *R*; results were obtained by simulating a breeding program. For a single trait, and assuming the candidates have the same amount of information and are identically distributed, *R* could be expressed in terms of the selection differential $(S=\mu_{S}-\mu_{1}$, the difference between the mean of the selected individuals, $\mu_{S}$, and the mean of the original population, $\mu_{1}$) multiplied by the heritability ${(h}^{2})$. Thus, *R=*$h^{2}$*S*, and when $h^{2}\to 1,$*R*→*S* (maximum expected response to selection, minimum expected loss in the decision of which candidates to select based on our breeding goals), whereas when $h^{2}\to 0$, *R ≪ S* (minimum expected response to selection, maximum expected loss)*.* The BDT methodology proposed by Villar-Hernández *et al.* (2018) considers the variance-covariance matrix between traits and the trait mean while minimizing the posterior expected distance between the distribution of the offspring of the selected individuals (distribution of the candidates) and the distribution of the selected individuals (distribution of the selected parents), and therefore maximizing the expected response to selection (*R*) given the phenotypic, genotypic and genomic information at hand. Minimizing the distance between the distribution of the parental candidates and the progeny distribution increases the accuracy of selection (assuming equal selection intensity).

Multiple trait selection is a concern addressed by animal and plant breeding in the past (Smith 1936; Hazel 1943; Henderson and Quass 1976) and also in the era of GS (Sun *et al.* 2017; Montesinos-López *et al.* 2019; Neyhart *et al.* 2019; Lenz *et al.* 2020). Multi-trait selection models are promising because they have the potential to increase the accuracy of GEBV (given that they use information about genetically correlated traits), especially in the presence of low heritability traits (Jia and Jannink 2012; Guo *et al.* 2014; Ward *et al.* 2019). Also, the improvement of genetic stocks requires considering multiple traits simultaneously because economic value and net genetic merits depend on all traits (Falconer and Mackay 1996).

The selection of multi-traits can be facilitated by ranking lines based on a single number; for example, genomic selection indices (SI) score lines based on a weighted average of GEBV, and then select those lines with high scores (Cerón-Rojas and Crossa 2020). The approach of Villar-Hernández *et al.* (2018) ranks the lines based on the posterior expected loss (PEL, a single number) given our breeding goals, in terms of the mean and the genetic variance-covariance matrix. Thus, those candidates whose distributions are closer to the theoretical parental distribution will have the lowest expected loss value (high *R*), and the decision should be to advance those lines given that they reach the desired mean and keep the genetic variance as much as possible (high $h^{2}$).

As described in Villar-Hernández *et al.* (2018), the LF is the vehicle to go from the action space (candidate lines) to the resulting space (selected lines) given a Bayesian action (an action that guarantees a minimum PEL given our preferences). Of the three multivariate LFs used by Villar-Hernández *et al.* (2018), the Kullback–Leibler (KL) LF is easier to understand. We can compute the KL distance between two multivariate normal distributions, one of them truncated in a *t*-dimensional vector $y_{c}$ (reflecting the breeder’s preference for high or low phenotypic values, $y_{c}\;$of a length equal to the number of traits). The KL metric implies that the distance between both distributions decreases when the phenotypic (***P***) and genotypic variance-covariance (***G***) matrices tend to explain the same amount of variation between traits, *i.e.*, $GP^{-1}=I$ (a quantity similar to narrow-sense heritability in a single-trait setting). Depending on the trait, the KL metric employs the divergence criterion, which induces less penalty for those lines that have more density (probability) to the right of censoring values ($y_{c}$) (increasing BVs) or more density to the left of censoring values ($y_{c}$) (decreasing BVs).

Similar interpretations of other LFs, Energy Score and MALF can be found in Villar-Hernández *et al.* (2018). The three LFs were derived and described based on univariate and multi-trait heritability, the response to selection, and the selection differential.

The advantage of the Villar-Hernández *et al.* (2018) method is that while minimizing the LF, the response to selection is maximized, considering uncertainty throughout the full posterior predictive distributions, and not only based on punctual estimates. Although Villar-Hernández *et al.* (2018) presented simulated and real data, they did not present extensive practical applications. Therefore, based on the previous considerations, the main objective of this research is to show the practical details when applying the BDT in a real GS prediction based on quantitative genetic concepts in breeding decisions. We used two extensive datasets (multi-traits from 766 and 320 wheat lines) in which we applied 10% selection intensity (a value commonly used in GS-assisted breeding) according to the minimum PEL criterion.

## Materials and methods

### Experimental datasets

#### Dataset 1 (Elite wheat lines)

This dataset comprises information of 766 wheat lines at the Norman E. Borlaug Experiment Station (Ciudad Obregon, Sonora, Mexico). The traits are DTHD (days to heading), DTMT (days to maturity), Height (plant height), and GY (grain yield). The correlations between traits are: 0.84 for DTHD and DTMT, 0.01 for DTHD and GY, 0.2 for DTHD and Height, -0.06 for DTMT and GY, 0.14 for DTMT and Height, and 0.24 for GY and Height (Table 1A). Genotypic information is available in the form of the Genomic Relationship Matrix $G_{\left(766\;\times \;766\right)}$ (obtained from centered and standardized marker data). Both phenotypic and genomic data were previously used in Montesinos-López et al. (2019) and can be found in https://data.cimmyt.org/data set.xhtml? persistentId=hdl : 11529/10548141.

**Table 1:** Phenotypic correlations between the four traits in Dataset 1 [days to heading (DTHD), days to maturity (DTMT, grain yield (GY) and plant height (Height)] and between the four traits in Data set 2 [grain yield (GY), thousand-kernel weight (TKW), Zn content in the grain (GZnC) and Fe content in the grain (GFeC)]

(A) **Data set 1**				
	DTHD	DTMT	GY	Height
DTHD	1.00	**0.84**	0.01	0.20
DTMT	—	1.00	-0.06	0.14
GY	—	—	1.00	**0.24**
Height	—	—	—	1.00

**(B)** **Data set 2**				

	GY	TKW	GZnC	GFeC

GY	1.00	**0.204**	0.014	0.04
TKW	—	1.00	0.017	**0.16**
GZnC	—	—	1.00	**0.26**
GFeC	—	—	—	1.00

#### Dataset 2 (Wheat biofortification)

The data comprise 320 spring wheat lines evaluated in 2014 at the Norman E. Borlaug Experiment Station (Ciudad Obregon, Sonora, Mexico). Four traits were measured in each line: GY (grain yield), TKW (thousand-kernel weight), GZnC (zinc concentration in the grain) and GFeC (iron concentration in the grain). All traits were positively correlated: 0.204 between GY and TKW, 0.16 for TKW and GFeC, 0.26 for GZnC and GFeC, 0.014 for GY and GznC, 0.017 for TKW and GZnC, and 0.04 for GY and GFeC (Table 1B). The genomic information is composed of 24,497 centered and scaled DaRT markers from which we calculated the Genomic Relationship Matrix **G**, as described in the previous dataset. A full description of phenotypic and genotypic information was given in Velu et al. (2016), and both datasets can be found at the link below.

### Data and software availability

The two datasets used in this study and the codes for running the proposed models can be found at the following link http://hdl.handle.net/11529/10548420.

### Direction of improvement in the datasets

In Dataset 1, selection of lines with low GEBVs for DTHD, DTMT, and Plant Height is required; thus, improvement focuses on the decreasing direction (–) of the trait. In contrast, for trait GY, progress lies in the increasing direction (+), *i.e.*, breeders want those lines with high GEBVs for GY. In Dataset 2, improvement of all traits under consideration lies in the positive (increasing) direction, *i.e.*, lines with high GEBVs.

### Loss function as a mechanism for parental selection

In general, the LF should reflect the distance/divergence of two probability distributions. The expected distance (loss of information) will be minimal if the two distributions approach each other, and the distance will be zero if both distributions are identical. Therefore, we can measure how close the distribution of the candidates is to the truncated parental distribution.

#### Univariate KL loss function

We first describe the single-trait case based on the idea of truncation selection. Let $y_{c}$ be a scalar. Lines with EBV above $y_{c}$ are the selected ones. The population from which we select the best lines is the base population (with some mean $\mu_{1}$ and some variance $\sigma^{2}$). For most quantitative traits, normal distribution is assumed for the base population, *i.e.*, $Y∼N(\mu_{1},\sigma^{2})$; then the selected lines (parents in the following generation) formally follow a truncated distribution, *i.e.*, $Y_{s}∼N_{T}\left(\mu_{1},\sigma^{2},a=y_{c},\;b=\infty \right)$ using the formal definition of a truncated distribution, but for simplicity, hereinafter we will denote $Y_{s}∼N_{T}\left(\mu_{1},\sigma^{2},y_{c}\right)$. Note that truncated normal distribution is a function of three parameters: ($\mu_{1},\sigma^{2},y_{c}$) and the mean of BVs after truncation occurs is $\mu_{S}=\mu_{1}+\sigma \left(\frac{\phi ((y_{c}-\mu_{1})/\sigma )}{1-\Phi ((y_{c}-\mu_{1})/\sigma )}\right)$, where ϕ and Φ denote the probability density function and the distribution function of standard normal density, respectively. Also, note that this occurs within the same generation.

After crossing the selected lines, there is an offspring population that has some distribution with mean ($\mu_{2}$) hopefully greater than the mean of the base population ($\mu_{2}>\mu_{1}$) and with some variance (we expect that it will be approximately equal to the variance of the base population in order to maintain the genetic diversity). We assume that the offspring population is also approximately normal, *i.e.*, $Y_{o}∼N(\mu_{2},\sigma^{2})$. Then, using this idea, we can construct a metric that quantifies the distance between the distribution of the truncated (parental) population and the distribution of the offspring (candidates for selection) population, such that the candidates that guarantee maximum genetic progress are those that guarantee minimum distance between the two distributions; this occurs between generations. With this idea in mind, we can construct any metric, for example, based on the Kullback Leibler (KL) loss function or any other divergence function. It is important to note that KL distance can be calculated whatever the distribution of the parents and the candidates is. When normality is assumed, KL has an analytical expression, and otherwise can be approximated with numerical or simulation methods.

Univariate KL, as presented below in Equations 1a–1c, appears when we calculate and simplify the expectation of the log ratio between the theoretical parental distribution and the candidate distribution with respect to the base distribution:

(1a) $$D_{KL}\left(F_{Y_{o}},F_{Y_{s}}\right)=\int_{y_{c}}^{\infty }\log\frac{N_{T}\left(\mu_{1},\sigma^{2},y_{c}\right)}{N\left(\mu_{2},\sigma^{2}\right)}N_{T}(\mu_{1},\sigma^{2},y_{c})dy$$

(1b) $$\;=\log\frac{1}{\mathrm{Pr}\left(y>y_{c}\right)}+\frac{1}{2}\left\{\frac{{\left(S-R\right)}^{2}}{\sigma^{2}}-i^{2}\right\}$$

(1c) $$\;=\log\frac{1}{\mathrm{Pr}\left(y>y_{c}\right)}+\frac{1}{2}\left\{i^{2}(h^{2}(h^{2}-2))\right\}.$$

In the previous equations, the divergence measured is between $F_{Y_{o}}$ and $F_{Y_{s}}$, where $F_{Y_{o}}$ denotes the distribution function of $Y_{o}\;$(random variable representing the phenotypic values of the offspring/candidate with mean $\mu_{2}$) and $F_{Y_{s}}$ represents the distribution function of $Y_{s}$ (random variable denoting the phenotypes of selected lines obtained based on truncation selection with mean $\mu_{s}$). Thus, $S=\mu_{S}-\mu_{1}$ is the selection differential, $R=\mu_{2}-\mu_{1}$ is the selection response, and the standardized selection differential i=S/σ is the selection intensity. The second term on the right-hand side of Equation 1b implies that when *R* approaches *S* (while the selection intensity stays the same), the divergence between the truncated distribution and the candidate’s distribution decreases and the genetic gain increases. That is, $D_{KL}\left(F_{Y_{o}},F_{Y_{s}}\right)$ depends on the intensity of selection (which is assumed fixed) and is a decreasing function of $h^{2}$ (1c).

**Figure A1:**
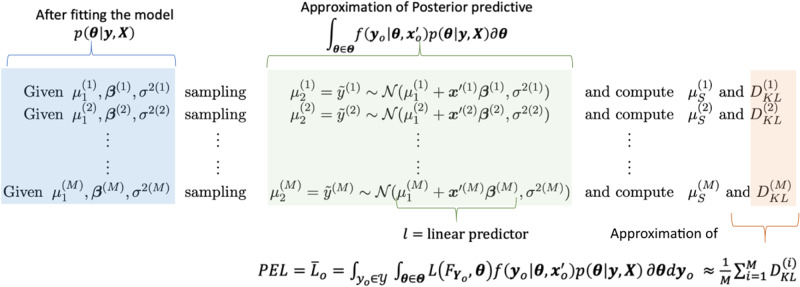
Computation of the posterior distribution of one line and approximation of the posterior expected loss (PEL) using MCMC samples of the posterior distribution of the model’s parameters after fitting the model using the Kullback–Leibler (KL) loss function. The number inside ( ) denotes the iteration of the procedure to approximate the posterior predictive distribution and PEL.

**Figure A2:**
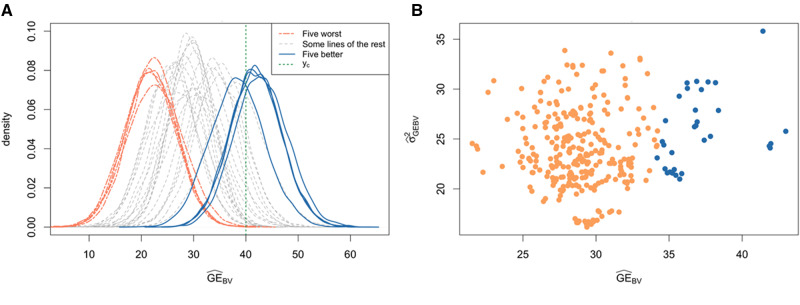
(A) Posterior distributions of the 5 best and the 5 worst lines and some of the rest of the lines for trait GFeC in Dataset 2. (B) Pair-wise plot of GEBV and posterior variance for all lines in Dataset 2; the blue dots represent the 32 selected lines according to the minimum posterior expected loss (PEL) using the Kullback–Leibler distance.

Note that the KL distance is not specific to normal distribution and can be applied to any pair of distributions. Appendix 1 (Figures A1 and A2) shows a step-by-step explanation of a Bayesian decision approach using the KL distance metric to measure the distance between any pair of distributions (candidate distribution and truncated distribution).

**Figure 1:**
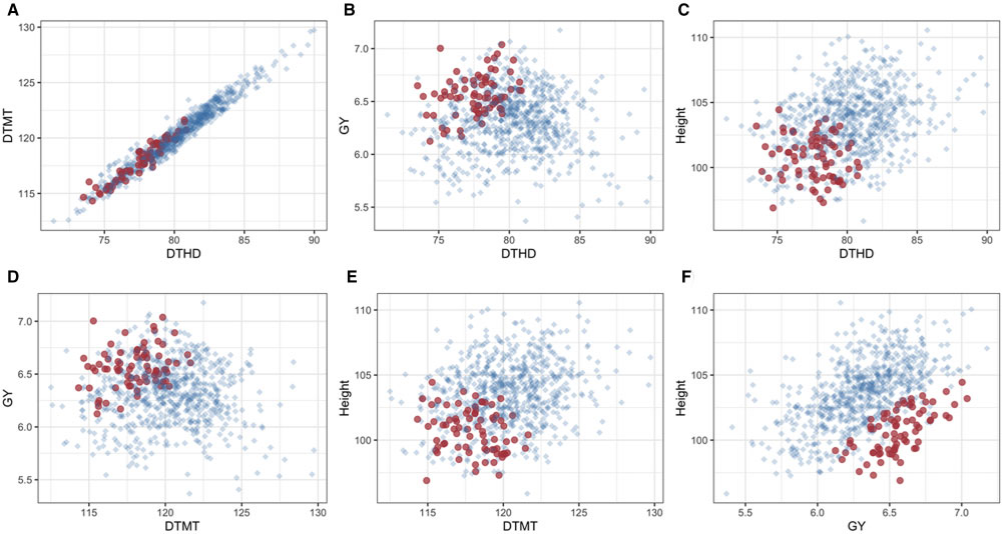
Pair-wise plots of GEBVs for traits in Dataset 1 using KL loss function. In (A) DTHD(–) and DTMT(–); (B) DTHD(–) and GY(+); (C) DTHD (−) and Height (−); (D) DTMT(–) and GY(+); (E) DTMT(+) and Height(–); (F) GY(+) and Height(–). The desired direction of improvement is illustrated as (+) = increasing direction of the trait, and (–) = decreasing direction of the trait. Red dots represent 10% of the selected lines with minimum PEL.

**Figure 2:**
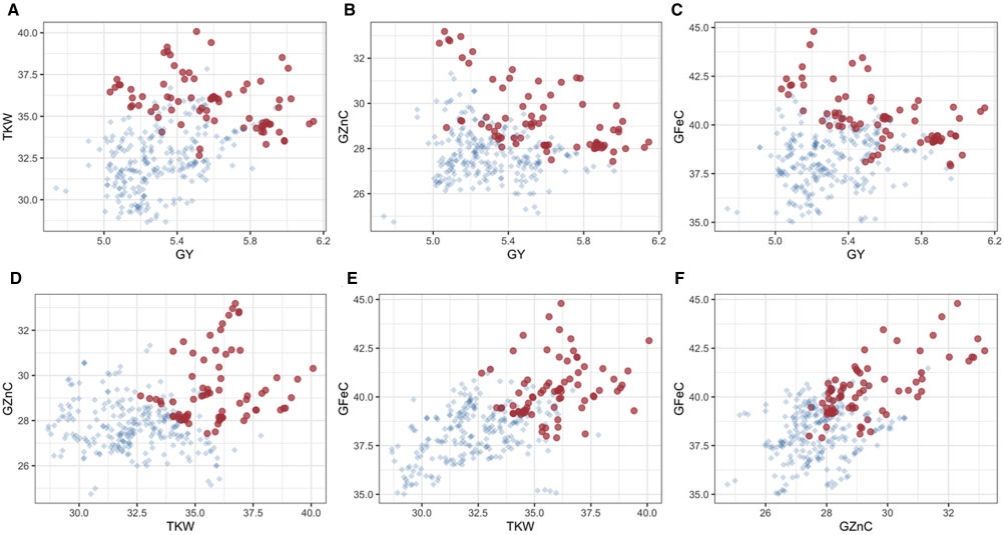
Pair-wise plots of GEBVs for traits in Dataset 2 using KL loss function. In (A) GY and TKW; (B) GY and GZnC; (C) GY and GFeC; (D) TKW and GZnC; (E) TKW and GFeC; (F) GZnC and GFeC. The desired improvement for all traits is in the increasing direction, *i.e.*, we wish to increase the GEBVs of all four traits. Red dots represent 10% of the selected lines with minimum PEL.

#### Multivariate KL loss function

The idea of truncation selection can be extended to multi-traits where the base population, the parental individuals, and the offspring population follow multivariate distributions. The parental distribution is a truncated distribution in a vector $y_{c}$ (of a length equal to the number of traits). Assuming multivariate normal distributions and using the KL distance, the formulation of the multivariate KL LF is expressed in Equation 2a. The Multivariate Truncated Normal (TMVN) distribution has mean vector $\mu_{1}$, phenotypic variance-covariance matrix P, and truncation vector $y_{c}$. The Multivariate Normal (MVN) distribution has a mean vector $\mu_{2}$ and the same P as the base population. After simplification, KL reduces to (2 b):

(2a) $$\;D_{KL}\left(F_{Y_{o}},F_{Y_{s}}\right)=\int_{y_{c}}^{\infty }\log\frac{\mathrm{TMVN}(\mu_{1},P,y_{c})}{\mathrm{MVN}(\mu_{2},\;P)}T\mathrm{MVN}(\mu_{1},P,y_{c})dy$$

(2b) $$\;=-\log\left(z\right)+\frac{1}{2}S^{'}\left[{\left(I-GP^{-1}\right)}^{'}P^{-1}\left(I-GP^{-1}\right){-P}^{-1}\right]S.\;$$

Upon inspecting Equation 2b, when phenotypic (P) and genotypic variance-covariance (G) matrices tend to explain the same amount of variation and similar association between traits, then $(I-GP^{-1})\to 0$, *i.e.*, the distance between the truncated distribution and the candidate/offspring distribution tends to decrease. The product matrix $GP^{-1}=h^{2}\;$is equivalent to multi-trait heritability (the ratio of the genetic variance in the numerator and the phenotypic variance in the denominator); thus, when $GP^{-1}=I$, the heritability of the traits approaches 1; then R=S and the mean of the offspring/candidate distribution is equal to the mean of the truncated distribution, $\mu_{2}=\mu_{s}$. The multivariate KL LF contains the term $–\log\left(z\right),$ which indicates that the joint probability of multivariate BVs that is > $y_{c}$ has less penalty. As already mentioned for the single trait, the KL distance is not specific to normal distribution.

#### Another two multi-trait loss functions: MALF and Energy Score

We can use other metrics, not only the KL. Two simple and easy to implement LFs are the Energy Score and the Multivariate Asymmetric Loss Function (MALF). Both measures are generalizations of the single-trait selection fully discussed in Villar-Hernández et al. (2018). Brief theoretical descriptions of the Energy Score and MALF are given in Appendix 2.

### Fitting the multi-trait mixed model and evaluating the posterior expected loss function

To conduct selection through the Bayesian decision framework within genomic-enabled prediction of multiple traits, three steps are required: Step1: training a regression model using available phenotypic multivariate data and genotypic records (training population); Step 2: approximate posterior predictive distributions of each candidate assuming the same sampling model as in step 1; Step 3: approximate the PEL for each candidate so that we can identify the lines with minimum PEL that fulfill the desired selection intensity.

In our case, the multiple trait regression model used in step 1 was the following:

(3) y1y2y3y4=1nμ11nμ21nμ31nμ4+Z10000Z20000Z30000Z4g1g2g3g4+ϵ1ϵ2ϵ3ϵ4, 

where ${(y}_{1},y_{2},y_{3},y_{4})'$ is the vector (or vectors) of phenotypic values for traits 1,2,3 and 4, respectively, $\mu_{1},\mu_{2},\mu_{3}$ and $\mu_{4}$ are the means for each trait, ${(g}_{1},g_{2},g_{3},g_{4})'$ is the vector of genomic values of each line for the four traits, $Z_{1},Z_{2},Z_{3}$ and $Z_{4}$ are the corresponding design matrices for random effects, and ${(\epsilon }_{1},\epsilon_{2},\epsilon_{3},\epsilon_{4})'$ is the vector of random residuals for all traits. Assuming that ${(g}_{1},g_{2},g_{3},g_{4})'∼\mathrm{MVN}(0,\;\Sigma_{g}⊗G)$, where

$$\Sigma_{g}=\left(\begin{array}{cccc} \sigma_{g_{1}}^{2} & \sigma_{g_{12}} & \sigma_{g_{13}} & \sigma_{g_{14}}\\ \sigma_{g_{21}} & \sigma_{g_{2}}^{2} & \sigma_{g_{23}} & \sigma_{g_{24}}\\ \sigma_{g_{31}} & \sigma_{g_{32}} & \sigma_{g_{3}}^{2} & \sigma_{g_{34}}\\ \sigma_{g_{41}} & \sigma_{g_{42}} & \sigma_{g_{43}} & \sigma_{g_{4}}^{2} \end{array}\right)$$

is the variance-covariance matrix of genomic values for the four traits, and ${(\epsilon }_{1},\epsilon_{2},\epsilon_{3},\epsilon_{4})'∼\mathrm{MVN}(0,R⊗I)$ with $I_{n\times n}$ being the identity matrix, and

$$R=\left(\begin{array}{cccc} \sigma_{e_{1}}^{2} & 0 & 0 & 0\\ 0 & \sigma_{e_{2}}^{2} & 0 & 0\\ 0 & 0 & \sigma_{e_{3}}^{2} & 0\\ 0 & 0 & 0 & \sigma_{e_{4}}^{2} \end{array}\right)$$

the residual variance-covariance matrix. The model in (3) was fitted using the MTM R package (de los Campos and Grüneberg 2016, that is available at GitHub: https://github.com/QuantGen/MTM) and R programming language version 3.6.1 (R Development Core Team 2019). The diagonal matrix ***R*** assumes a null residual covariance matrix among environments that is seldom used in plant breeding and animal breeding to avoid an important increase in computing time.

After fitting the model given in Equation 3, we used outputs from the MTM R package to estimate the full posterior distributions of the model’s parameters using 10,000 MCMC (Markov Chain Monte Carlo) samples after discarding the 30,000 samples as burn-in and thinning at lag 5. We then approximated the posterior predictive distribution of each line considered as a candidate, using multivariate normal distribution as the sampling model. Finally, the LF is inserted in Equation 4 in order to approximate the PEL of each candidate

(4) $${\overset{-}{L}}_{o}=\int_{y_{o}\in Y}\;\int_{\theta \in \Theta }L\left(F_{Y_{o}},\theta \right)f\left(y_{o}|\theta ,\;x_{o}^{'}\right)p\left(\theta |y,X\right){\partial \theta dy}_{o},$$

where $p\left(\theta |y,X\right)$ represent the joint posterior distributions of the model’s parameters after fitting the model in step 1 (model in Equation 3), and $f\left(y_{o}|\theta ,\;x_{o}^{'}\right)$ denotes the multivariate normal distribution (the sampling model), $L\left(F_{Y_{o}},\theta \right)$ is the used LF (KL or Energy Score or MALF), and ${\bar{L}}_{o}$ is the PEL. Then, for each candidate *o*, Equation 4 has to be evaluated. Integrals cannot be evaluated analytically; instead we used approximation iterating at each realization of MCMC chains; then the integrals are reduced to summations and averaged over the total number of MCMC chains (for breeders and geneticists who are not familiar with MCMC theory and Bayesian statistics, we attached all the source codes used in this study). It is important to recall that both the regression model in (3) and the LF used, can be replaced with any multiple trait model and any LF, *i.e.*, the Bayesian decision approach is a general formulation. Here, we used three LFs that we think have a genetic sense.

It is important to note that the MTM R Package does not return posterior distributions of the model’s parameters (which are needed to approximate the posterior predictive distribution) as MCMC objects, but internally it does. Therefore, we downloaded the source code and modified a couple of lines to save them. In http://hdl.handle.net/11529/10548420 the modified source code is added to all the datasets for reproducibility of the results presented in this paper.

In this study, we used information on 767 lines in Dataset 1 and the 320 records in Dataset 2 to train the regression model; the aim was to select the best 10% of lines (76 lines selected from Dataset 1 and 32 lines selected from Dataset 2) with minimum PEL; thus, the entire set of lines were considered as candidates.

As previously mentioned, we need a threshold vector for the BDT approach in multi-trait selection. Thus, to analyze Dataset 1, we fixed $y_{c}\;=\;{\left(76_{q_{0.1}},\;116_{q_{0.1}},\;97_{q_{0.1}},\;7_{q_{0.9}}\right)}^{'}$ for traits DTHD(–), DTMT(–), Height(–) and GY(+), respectively. These values are lower than the simple averages 79.9 (DTHD), 120 (DTMT) and 103 (Height), given that improvement of these traits is in the decreasing direction. For trait GY, improvement is in the increasing direction, as 7 is greater than the average of phenotypic values of GY (6.3). Sub-indexes $q_{0.1}$ and $q_{0.9}$ denote 0.1 and 0.9 empirical quantiles of observed phenotypic values of the corresponding traits.

For Dataset 2, the desired direction for all traits is in the increasing direction, so we chose $y_{c}\;=\;(6,\;38,\;31,\;43)'$ for traits GY(+), TKW(+), GZnC(+), and GFeC(+), respectively. Each value in $y_{c}$ is greater than the simple average of the traits, 5.3 (GY), 33.1 (TKW), 28.1 (GZnC) and 38.6 (GFeC), and in all traits corresponds to 0.9 empirical quantile of observed phenotypic values. Note that the Bayesian decision formulation requires censoring on the right side of the parental distribution to reflect that improvement is in the positive direction, but in cases where the improvement is in the decreasing direction, we need a truncation on the left side of the distribution. A practical approach is to change the sign (multiplying by −1) of the GEBVs when evaluating the LF. This approach was used for traits DTHD(–), DTMT(–) and Height(–) in Dataset 1.

## Results

### Dataset 1

**Figure A3:**
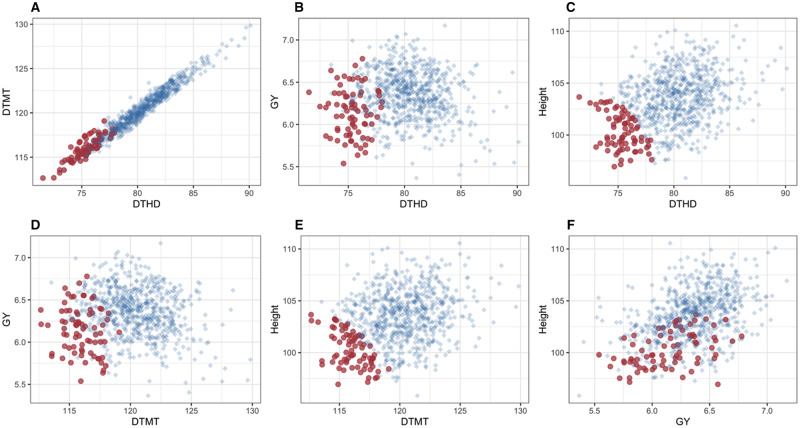
Pair-wise plots of GEBVs for traits in Dataset 1 using Energy Score. In (A) are plotted DTHD (–) and DTMT (–); (B) DTHD (–) and GY (+); (C) DTHD (–) and Height (–); (D) DTMT(–) and GY (+); (E) DTMT (+) and Height (–); (F) GY(+) and Height (–). The desired direction of improvement is illustrated as (+) = increasing direction of the trait, and (–) = decreasing direction of the trait. Red dots represent 10% of the selected lines with minimum PEL.

**Figure A4:**
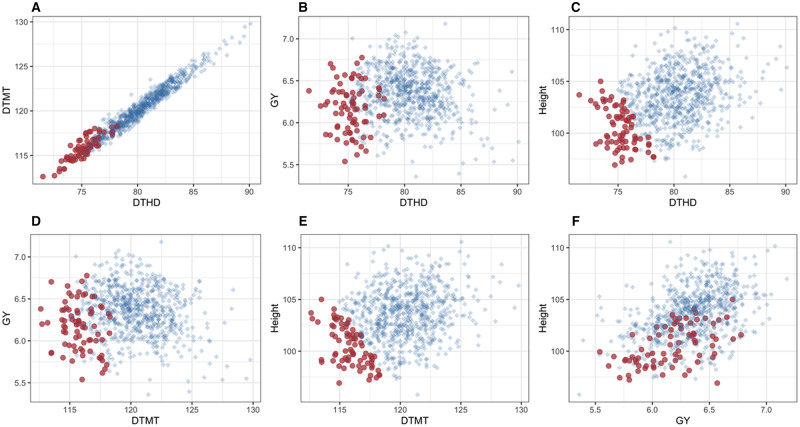
Pair-wise plots of GEBVs for traits in Dataset 1 using MALF. In (A) are plotted DTHD (–) and DTMT (–); (B) DTHD (–) and GY(+); (C) DTHD (–) and Height (–); (D) DTMT (–) and GY(+); (E) DTMT (+) and Height (–); (F) GY (+) and Height (–). The desired direction of improvement is illustrated as (+) = increasing direction of the trait, and (–) = decreasing direction of the trait. Red dots represent 10% of the selected lines with minimum PEL.

Figure 1 displays pair-wise plots of the GEBVs for the four traits in Dataset 1 obtained under the KL LF. Figure 1A plots traits DTHD(–) and DTMT(–), given that improvement is in the decreasing direction for both traits, and the 76 selected lines (red dots) are concentrated at low values of GEBVs for both traits; the phenotypic correlation between DTHD and DTMT is high: 0.84 (Table 1). Similar results are observed in Figure 1C for DTHD(–) and Height(–) and in Figure 1E for the pair of traits DTMT(+) and Height(–) with correlations of 0.20 and 0.14, respectively. Figure 1E depicts GEBVs of DTHD(–) and GY(+), where the red dots are concentrated in the top left quadrant of the picture, given that we need to increase the phenotypic values of GY and, at the same time, decrease the value of DTHD. The same is shown in Figure 1D for the pair of traits DTMT(–) and GY(+), and finally, in Figure 1F, for GY(+) and Height(–), the selected lines are concentrated in the right bottom quadrant and have high GEBVs of GY and small values of Height. Phenotypic correlations of GY with the other traits were negligible, except with plant height (0.24) (Table 1). These lines are the best according to the KL loss, which considers improvement in all traits, in either the increasing or decreasing direction. Similar plots were obtained using the Energy Score (Figure A3, Appendix 3) MALF LFs (Figure A4, Appendix 3), both of them with similar interpretations.

Table 2 shows the values of the best five lines and the worst lines ranked based on the value of the three LFs, KL, Energy Score and MALF, for Dataset 1. Table 2 also shows, for each trait and for each of the five top ranking wheat lines and the worst five lines, the GEBVs and the PEL under each LF. Values of PEL were standardized to range from 0 (minimum loss) to 1 (maximum loss). The values inside () represent the rank of each line in the top 5 lines and in the worst 5 lines. Note that line 141 was ranked at the top (minimum PEL) for the three LFs. The reason for this is that it has low values of DTHD(–), DTMT(–) and Height(–), and although it does not have the maximum value of GY(+), it has a relative high GEBV (6.57 ton/ha for trait GY); thus it is reasonable that it is ranked first. Wheat line 635 with the highest GEBV of 6.8 tons/ha for GY(+) ranked five based on the KL LF; this is because the GEBVs for the other traits were not so low; the other four top lines (141, 232, 210, and 334) based on KL criteria had the lowest values for the other three traits DTHD, DTMT and Height; these lines gave slightly lower grain yield (6.6, 6.5, 6.6, and 6.7 tons/ha) than the line ranked five (635) based on KL criteria.

**Table 2:** Dataset 1

LINE	GEBV				Posterior expected loss		
	**DTHD**	**DTMT**	**GY**	Height	KL	Energy	MALF
**Top five lines**							
141	74.70	114.92	6.57	96.89	0 (1)	0 (1)	0 (1)
232	76.54	117.01	6.54	99.45	0.068 (2)	—	—
210	77.54	118.80	6.58	98.27	0.072 (3)	—	—
334	77.46	118.75	6.70	99.86	0.077 (4)	—	—
635	76.27	116.44	6.78	101.54	0.078 (5)	—	—
551	73.01	113.53	5.86	99.03	—	0.006 (2)	0.002 (2)
553	73.27	113.38	5.84	99.21	—	0.017 (3)	0.013 (3)
15	74.17	115.05	5.95	98.68	—	0.023 (4)	0.033 (4)
554	73.84	114.25	5.82	99.04	—	0.026 (5)	0.041 (5)
**Bottom five lines**							
547	89.03	127.52	6.21	107.15	0.805 (762)	0.911 (763)	0.929 (764)
753	85.72	125.03	6.16	110.56	0.916 (763)	—	—
485	87.90	127.46	5.53	105.31	0.959 (764)	—	—
351	89.98	129.73	5.78	106.36	0.995 (765)	1 (766)	1 (766)
478	88.51	128.33	6.38	106.72	—	0.903 (762)	0.907 (762)
320	89.73	129.61	5.55	104.24	1 (766)	0.943 (764)	0.927 (763)
546	89.54	128.39	6.61	108.45	—	0.98 (765)	0.98 (765)

The five best lines and five worst lines based on posterior expected loss (PEL), under KL, Energy Score and MALF loss functions. Summaries are given for GEBVs for all traits in Dataset 1. Values of PEL were standardized to vary from 0 (minimum) to 1 (maximum). Values in *(*) represent the rank of the line with respect to each loss function.

The other two LFs, Energy Score and MALF, except for line 141, selected another set of wheat lines in the top five, with lower values of the traits than the wheat lines selected by the KL LF. Regarding grain yield (+), the top lines selected under Energy Score and MALF had lower grain yield than those selected by the KL criterion; however, the Energy Score and MALF criteria selected lines with lower values for traits DTHD(–), DTMT(–), and Height(–) than the KL criteria. As for the worst five lines, both criteria selected similar lines. Although only line (141) was selected under all LF criteria, in the 76 lines selected to be parents of the next generation for Dataset 1, KL *vs* Energy Score selected 30.26% of the same lines, KL *vs* MALF selected 31.58% of the same lines, and Energy Score vs MALF selected 92.11% of the same lines. Of the five worst lines based on PEL, we can see that they have high values for traits DTHD, DTMT and Height, but it was difficult to find lines with low values for trait GY.

Table A1 (Appendix 3) shows the posterior variance of each line in the five best and the five worst lines for each LF applied to Dataset 1. Regarding the variances of the posterior predictive distribution of the top five lines, they were not very different for the four traits for all LFs. Of the top five lines based on KL only, two had the highest posterior variance (210 and 635); the line with the highest GEBV for trait GY (6.8 tons/ha) (635) (ranked 5 based on KL) had intermediate variance for the four traits.

In summary, for Dataset 1, the KL LF gave similar importance to all traits. In contrast, the Energy Score and MALF gave better performance in three of four traits (DTHD, DTMT and Height) leaving the GY trait as less important. In terms of the posterior variance, these differences were negligible. The LF approach should help breeders to decide based not only on the GEBV values per se of the parent to be selected, but also on the level of uncertainty according to the Bayesian paradigm.

### Dataset 2

**Figure A5:**
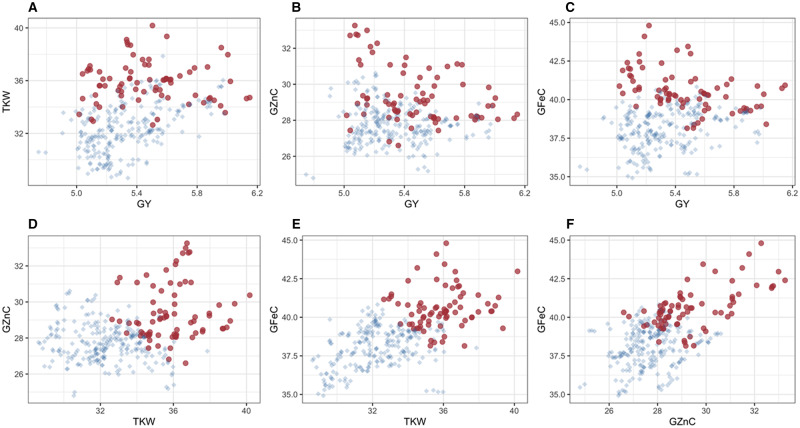
Pair-wise plots of GEBVs for traits in Dataset 2 using Energy Score. In (A) are plotted GY and TKW; (B) GY and GZnC; (C) GY and GFeC; (D) TKW and GZnC; (E) TKW and GFeC; (F) GZnC and GFeC. The desired improvement for all traits is in the increasing direction, *i.e.*, we wish to increase the GEBVs of all four traits. Red dots represent 10% of the selected lines with minimum PEL.

**Figure A6:**
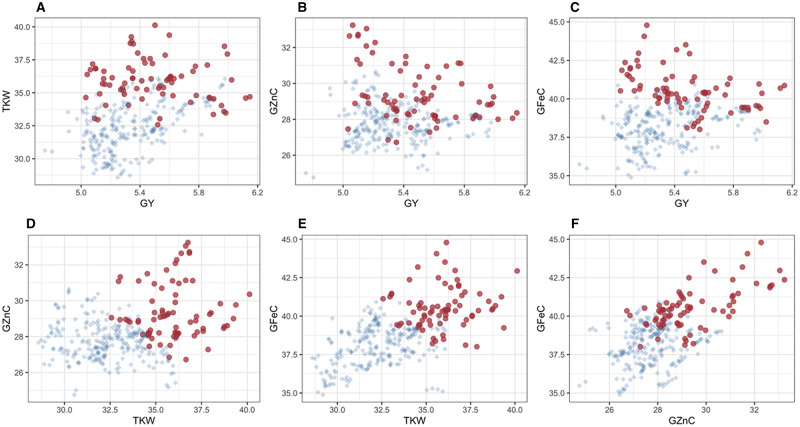
Pair-wise plots of GEBVs for traits in Dataset 2 using MALF. In (A) are plotted GY and TKW; (B) GY and GZnC; (C) GY and GFeC; (D) TKW and GZnC; (E) TKW and GFeC; (F) GZnC and GFeC. The desired improvement for all traits is in the increasing direction, *i.e.*, we wish to increase the GEBVs of all four traits. Red dots represent 10% of the selected lines with minimum PEL.

The phenotypic correlations among traits for Dataset 2 are shown in Table 1. Figure 2 displays a pair-wise plot for every combination of the posterior mean of the GEBV of the four traits. In this scenario, we are interested in increasing genetic gain in the positive direction for all traits. Therefore, the points representing selected individuals are in the top-right corner of the pair-wise plots for correlated traits. Those lines represented by red dots should be the ones breeders select to make crosses and move to the next improvement cycle, thereby assuring simultaneous increase in genetic gains (response to selection) in the four traits. Similar plots were obtained using the Energy Score (Figure A5, Appendix 3) and MALF LFs (Figure A6, Appendix 3), both of them with similar interpretation as in the KL LF.

The multi-trait values of the top five lines and the worst five lines ranked based on the three LFs, KL, Energy Score and MALF, for wheat Dataset 2 are shown in Table 3. In this case, wheat line 177 had the highest GEBV (5.97 tons/ha) for GY and was ranked in 1st place by KL LF; the other four top lines based on KL criteria also had high values of GEBV for TKW, GZnC and GFeC traits; the means of traits GY, TKW, GZnC and GFeC were 5.4, 33.2, 28.1 and 38.7, respectively, and all GEBVs for all traits had values greater than the mean. The other two LFs, Energy Score and MALF, selected different sets of wheat lines than those identified by KL; they also had lower values for GY (and higher for the other traits) than those wheat lines selected by the KL LF. As already mentioned, regarding grain yield, the top lines selected under Energy Score and MALF had lower grain yield than those selected by the KL criteria (lower than 5.5 tons/ha). However, the Energy Score and MALF criteria selected lines with higher values for traits GZnC and GFeC than the KL criteria. Only line 64 was selected for all LFs in this dataset in the top 5 best; KL ranked it as 4, but Energy Score and MALF ranked it as 1. Similarities in selected lines are depicted in Figure 2, A–F, Figures A5, A–F (Appendix 3) and Figures A6, A–F (Appendix 3) for all LFs for the 32 lines selected to be parents of the next generation, and are confirmed by the percentage of lines selected; thus, KL vs Energy Score selected 85.5% of the same lines, KL vs MALF 86.8%, and Energy Score vs MALF 96%. Regarding the five worst lines, all LFs identified the same lines, all of them with GEBVs less than the mean.

**Table 3:** Dataset 2

LINE	GEBV				Posterior expected loss		
	**GY**	**TKW**	**GZnC**	GFeC	KL	Energy	MALF
**Top five lines**							
177	5.97	38.52	29.90	40.91	0 (1)	—	—
202	5.78	36.94	31.11	41.25	0.002 (2)	—	—
201	5.76	36.57	31.13	40.92	0.018 (3)	—	
64	5.51	40.08	30.31	42.89	0.034 (4)	0 (1)	0 (1)
178	6.01	37.88	29.20	40.33	0.044 (5)	—	—
35	5.21	36.18	32.29	44.80	—	0.025 (3)	0.022 (2)
38	5.15	36.61	32.96	42.99	—	0.022 (2)	0.035 (3)
211	5.06	36.73	33.19	42.37	—	0.037 (4)	0.053 (4)
213	5.08	36.89	32.83	42.03	—	0.045 (5)	0.074 (5)
**Bottom five lines**							
232	5.01	29.29	26.67	35.49	0.872 (312)	0.93 (312)	0.95 (312)
234	5.09	29.21	26.42	35.02	0.89 (313)	0.988 (315)	0.996 (314)
233	5.08	28.92	26.44	35.09	0.902 (314)	1 (316)	1 (316)
72	4.74	30.70	25.00	35.70	0.986 (315)	0.935 (313)	0.97 (313)
73	4.79	30.51	24.75	35.51	1 (316)	0.957 (314)	0.996 (315)

The five best lines and five worst lines ranked based on posterior expected loss (PEL), under KL, Energy Score and MALF loss functions. Summaries are given for GEBVs for all traits in Dataset 2. Values of PEL were standardized to vary from 0 (minimum) to 1 (maximum). Values in *(*) represent the rank of the line with respect to each loss function.

Table A2 (Appendix 3), shows, for each trait, the variance of the posterior predictive distribution of the best 5 and the worst 5 wheat lines based on their respective LFs, and their ranking based on PEL. Regarding the variances of the posterior predictive distribution of the top five lines, they were not very different for the four traits based on KL criteria. Of the top five lines based on KL only, two had the highest posterior variance (177 and 201) for trait GY; line 202 had the highest posterior variance for trait GZnC (10.53) and line 64 had high genetic variance for trait GFeC.

In summary, for this dataset, results show that on average the LFs performed similarly, in terms of the posterior mean and the posterior variance of the selected individuals.

## Discussion

The main objective of this study was to present practical examples of how the GS via LF concepts and BDT of Villar-Hernández *et al.* (2018) can be used in candidate selection on two extensive datasets from which we wish to identify the 10% best performing individuals according to LF criteria, and advance them to the next generation of random mating. Based on this, we believe that the proposed multi-trait decision theory gives a clear interpretation of quantitative genetic and plant breeding methods because it selects the lines that maximize the response to selection of multi-traits by minimizing the LF (which, in turn, is a function of the heritability, selection differential and multi-trait phenotypic and genotypic covariance matrices).

To perform GS selection using a decision theory approach, the following steps are required: (1) training a multi-trait mixed regression model with genomic and phenotypic data, (2) approximating the posterior predictive distribution of each parental candidate using genomic information and the trained model, (3) calculating the PEL via MCMC approximation, and (4) selecting the best lines with minimum PEL according to the desired selection intensity.

The central part of the decision theory approach is the concept of an LF reflecting the breeder’s preferences for the best performing parental candidates for selection. By minimizing the PEL, we maximize the genetic progress in all traits considered (maximization of the net genetic merit of individual lines), understanding that genetic progress is a compromise between increasing/decreasing (depending on the desired direction of improvement) BV (or GEBV) for all traits in successive selection cycles with the lowest possible loss of genetic diversity.

The use of LF methodology raised some natural questions; for example, how to reflect breeders’ preference for high/low phenotypic values, or what the best values of $y_{c}$ (threshold vector) are. The proposed LF can be studied by incorporating inbreeding and co-ancestry information, and by extending the LF concept with non-Gaussian traits (Poisson, Binomial, etc.). The LFs facilitate the selection of multi-trait scoring in a single metric (a scalar) for each candidate line to be a parent of the next generation. The selection of multi-traits is important because the net genetic merit and the economic value usually depend more on some traits than on others. In general, genomic-enabled prediction multi-trait models have become more useful than single-trait models because trait correlation information can be exploited to increase the prediction accuracy of correlated traits. In fact, multi-trait selection occurs even using a single-trait selection approach; however, if selection is based on a multi-trait regression model (parametrized as mixed or not) and the BDT framework, the researcher is selecting the best performing individuals for all the traits together. The BDT is the ideal approach for correlated traits.

By formulating the multi-trait selection in GS as a Bayesian decision problem, all uncertainty/risk components such as uncertainty in model parameters (mean and variance) and uncertainty in the effects of molecular markers are simultaneously considered in the LF when computing PEL. Furthermore, the LFs can be interpreted in terms of common concepts for geneticists such as heritability, and LFs are minimized when heritability increases, as was pointed out by Villar-Hernández *et al.* (2018). As a complement, LF concepts and selection through Bayesian decision is a well-established theory in statistics and its applications (Berger 1985, Ch4; Dawid 2007; Robert 2007, Ch2). Bayesian decision is a coherent way of selecting the “best parents/individuals” to advance in GS because the consequences of selection cannot be completely anticipated and uncertainty is contemplated in a unified framework. Expected loss theory assigns a quantitative loss to each possible decision, and then selects an action that minimizes the expected value of the resulting loss. This idea has proven to be a widely applicable description of rational behavior (Parmigiani and Inoue 2009).

In this study, we conducted a selection of parental individuals using the LF approach in two extensive real wheat datasets comprising four traits. In the first dataset (Dataset 1), the genetic progress of three of four traits was in the decreasing direction, and in one trait the progress needed to increase; however, correlations between traits were positive but low, except for two traits. In the second dataset (Dataset 2), the progress needed was in the increasing direction and correlations between traits were, in general, negligible. Results from both datasets indicate that all LFs performed similarly, although in Dataset 1 there was a minor advantage of Energy Score and MALF functions over the KL (Energy Score and MALF performed better than KL loss in three of four traits, but sacrificed one trait: GY). This small difference in favor of Energy Score and MALF may be explained by the fact that in Dataset 1 there are traits whose improvement directions are in the positive and negative sense, or that positive and negative correlations between traits are present. These cases were not present in Dataset 2. In terms of posterior variance, the three LFs performed similarly in both datasets, *i.e.*, sometimes LFs selected lines with high posterior variance, but other times they selected lines with low/medium posterior variance, but on average, percentual differences were insignificant. Additionally, it is important to note that Energy Score and MALF selected up to 96% of the same lines. In general, results from both datasets show that the lines with the highest GY values are not always those that will give less uncertainty and minimize the LF while maximizing the response to selection. The reason for this is that LFs weighted gains in all traits, not only GY; in Dataset 1, the best GY line ranked 5th under KL criteria and was not selected at all under Energy and MALF LF criteria. However, in Dataset 1, one line was selected as the best parent for the three LFs. In Dataset 2, the best GY line was the one with minimum KL value, but a line that was ranked 4th based on KL criteria with less GY than the one that ranked first was in fact selected as the best based on Energy and MALF criteria.

The use of Mean Squared Error, is in a sense, a quadratic distance that has its generalization in the LF named Continuous Ranked Probability Score (CRPS). The Energy Score LF, on the other hand, is a generalization of the CRPS LF in the multivariate context. Thus, on this respect the “mean squared error” is indeed included in the context of this study by means of the “Energy Score” LF.

### Some differences between the multi-trait Bayesian decision and selection indices

Both the multi-trait BDT and the SI theory are indeed related because they are based on estimations of P and G. However, differences can be pointed out. First, the main difficulty when using SI in plant breeding is determining a vector of economic weights, whereas when using the Bayesian decision approach, the truncated $y_{c}$ values for the truncated distribution are easy to determine for increasing or/and decreasing traits. The BDT framework uses complete posterior distributions of each candidates and not only punctual estimates as SI does. The SI maximizes the response to selection based on the estimation of the coefficients of the economic weights that maximizes the correlation between the index and the net genetic merits and thus maximizes the selection response, whereas the BDT employs the divergence between distributions, which, as already mentioned, induces less penalty for those lines that have more density to the right of censoring values ($y_{c}$) or put more density to the left of censoring values ($y_{c}$). The three LFs used here, were derived and described based on univariate and multi-trait heritability, response to selection, and selection differential.

Although economic weights are not necessary to implement selections indices, a relevant question would be how to implement these economical weight in the proposed LFs. Further research if required for comparing the multivariate LFs proposed in this study and the selection index theory and practice applied in breeding. Another comparison of the multivariate LF of the proposed method could be with a relevant methodology based on multi-objective optimization based approaches as in Akdemir *et al.* (2018).

## Conclusions

In this research we conducted multi-trait selection of the best performing individuals using two extensive real wheat datasets with four traits under selection, through the BDT framework and LF concept. The main objective was to show a practical application and clarify some doubts and omissions in explanations not covered in the proposal of the authors in a preceding work (Villar-Hernández *et al.* 2018) where three LFs were explained as mechanisms for conducting multi-trait selection: the KL, the Energy Score, and the MALF. After applying the methodology, we found that for our datasets, all LFs performed similarly, selecting a subset of lines that guarantees the greatest genetic progress of all traits, although for one dataset we found a small advantage of Energy Score and MALF over the KL loss (*i.e.*, in three of four traits in Dataset 1, the Energy Score and MALF reported greater gains than KL). In terms of genetic variance, the three LFs performed similarly in both datasets, *i.e.*, sometimes LFs selected lines with high variance, but other times they selected lines with low/medium variance, but on average, the perceptual differences in variance with respect to the variance of the whole population for the three LFs were insignificant. Selection using LFs has the potential to be effective in multi-trait selection in GS given that it summarizes all relevant genetic concepts such as heritability, response to selection and the structure of dependence between traits (correlation).

## Appendix 1: Single-trait detailed example

This appendix shows how to conduct a Bayesian decision approach to a case of single-trait selection. We considered only trait GFeC from Dataset 2, and the Kullback–Leibler distance. The KL metric can be used to measure the distance between any pair of distributions, not only normal distributions. In this step-by-step example, we used the KL metric for two normal distributions. For a single-trait scenario, the model is written as

$$y_{i}=\mu_{1}+\sum_{j=1}^{p}x_{ij}\beta_{j}+\epsilon_{i},$$

where $y_{i}$ denotes the phenotypic values of trait GFeC for individuals i=1,2,…,n, $\mu_{1}$ is the general mean, $x_{ij}$ has the genotypes of line i in the j-th molecular marker, $\beta_{j}$ is the associated coefficient for the j-th predictor, and $\epsilon_{i}$ is the residual term. If we assume that $\epsilon_{i}∼N(0,\sigma^{2})$, then the previous model is equivalent to $y_{i}∼N(\mu_{1}+x'\beta ,\sigma^{2})$. Note that $x'\beta =\sum_{j=1}^{p}x_{ij}\beta_{j}$ is the signal given by the genomic information.

One way to fit the previous model is using Bayesian Ridge Regression and imposing appropriate prior distributions on the model’s parameters ($\theta =[\mu_{1},\beta ,\sigma^{2}]'$ with $\beta =[\beta_{1},\ldots ,\beta_{p}]'$). Details are in the BGLR of Pérez-Rodríguez and de los Campos (2014). BGLR applies the Gibbs Sampler Algorithm to approximate full posterior distributions of each component of θ (Casella and George 1992**)**.

After fitting the model and discarding the first 20,000 samples and thinning at lag 5, we had M=8000 samples from stationary distributions of the model’s θ parameters. For this example of a single-trait case, we fixed $y_{c}=40.$ To obtain full posterior distributions of each of the 316 lines in Dataset 2, we used the same sampling model, *i.e.*, $y_{i}∼N(\mu_{1}+\sum_{j=1}^{p}x_{ij}\beta_{j},\sigma^{2})$, and approximated PEL as is described next. Figure A1 graphically shows how we computed the posterior predictive distribution and the KL loss for one line at each iteration of the MCMC samples; this process is repeated for each line in the candidate set, *i.e.*, for the 316 lines.

After the previous steps, we obtained the PEL for each line, and ranked the lines from minimum to maximum PEL to identify the best 32 lines according to the LF criterion. Figure A2A shows the posterior distributions of the best five lines and the worst five lines according to the KL loss; some of the distributions of the lines not in the five best sets and not in the five worst sets are also displayed. Figure A2B shows on the *x*-axis the estimated GEBV, and on the *y*-axis, the posterior variance of each of the 320 lines in our dataset. After selecting by the LF criterion, the blue dots represent the individuals that were selected and the orange dots represent the individuals not selected. As can be observed, the selected lines have high values of GEBV and posterior variance values, which is not surprising because the analytical expression in Equation 1c (article) guarantees that. The procedure described above is easily extended to the multi-trait scenario, replacing the univariate sampling model for the multivariate normal distribution.

## Appendix 2: The Energy Score and MALF loss functions

The Energy Score is a multivariate loss function that comes from a well-known univariate loss function called Continuous Ranked Probability Score (CRPS) that is used in diverse applications (Hersbach 2000; Gneiting and Raftery 2007). The generalization of CRPS to multivariate scenarios was addressed by Székely and Rizzo (2013). For multi-trait GS purposes, the Energy Score is expressed as

(2.1) ESFYo,μs=EF‖Yo-μs‖-12EF‖Yo-Yo'‖

where ‖⋅‖ denote the Euclidean norm, $Y_{o}$ and $\mu_{s}$ were previously defined, and $Y_{o}’$ denotes an independent random vector with the same distribution as $Y_{o}$, i.e, $F_{Y_{o}}$.

Another multivariate loss function tested in this research is MALF (Komunjer and Owyang 2012), which is an asymmetric multivariate loss function. MALF comes from a very commonly used univariate loss function known in financial and actuarial literature as LinLin (Linear-Linear) Loss (Berk 2011). The MALF for multi-trait GS is expressed as

(2.2) $$L_{1}\left({F_{Y}}_{o},\mu_{s},\tau \right)=\left|e\right|+\tau^{'}e.$$

where $e={\left(\mu_{s}-\mu_{2}\right)}^{'}$ or, alternatively, expressed as $e=S-R=S\left(I-GP^{-1}\right)$ (note that e is a vector of length equal to the number of traits), τ is a vector of length equal to the number of traits that controls the degree of asymmetry and has support (-1<τ<1). The term $\left|e\right|=\left(\left|e_{1}\right|+\left|e_{2}\right|+\cdots +\left|e_{t}\right|\right)=\sum \left|e_{i}\right|$, *i.e.*, the $L_{1}-\mathrm{norm}$, whereas $e=(e_{1},e_{2},\ldots ,e_{t})'$ is the vector of deviations for trait 1,2,…,t. Note that when $(I-GP^{-1})\to 0$, the MALF is minimized, similar to KL loss.

## Appendix 3

**Table A1:** Dataset 1

LINE	Posterior variance				Expected loss		
	**DTHD**	**DTMT**	**GY**	Height	KL	Energy	MALF
**Top five lines**							
141	32.49	27.70	0.45	23.18	0 (1)	0 (1)	0 (1)
232	32.23	27.55	0.44	23.44	0.068 (2)	—-	—-
210	33.42	28.81	0.44	23.56	0.072 (3)	—-	—-
334	32.36	28.11	0.44	22.72	0.077 (4)	—-	—-
635	33.54	28.93	0.44	23.33	0.078 (5)	—-	—-
15	32.06	27.79	0.45	23.03	—-	0.023 (4)	0.033 (4)
551	32.71	28.20	0.46	23.54	—-	0.006 (2)	0.002 (2)
553	32.87	27.88	0.45	23.98	—-	0.017 (3)	0.013 (3)
554	32.66	28.05	0.44	23.41	—-	0.026 (5)	0.041 (5)
**Bottom five lines**							
547	32.29	27.74	0.43	23.25	0.805 (762)	0.911 (763)	0.929 (764)
753	32.02	27.58	0.45	23.61	0.916 (763)	—-	—-
485	33.39	28.87	0.44	23.36	0.959 (764)	—-	—-
351	32.12	27.49	0.44	23.02	0.995 (765)	1 (766)	1 (766)
320	31.50	27.42	0.44	22.83	1 (766)	0.943 (764)	0.927 (763)
478	32.88	28.13	0.45	23.03	—-	0.903 (762)	0.907 (762)
546	32.56	27.76	0.44	23.37	—-	0.98 (765)	0.98 (765)

Five best lines and five worst lines based on posterior expected loss (PEL), under KL, Energy Score and MALF. Summaries are given for posterior variances. Values of PEL were standardized to vary from 0 (minimum) to 1 (maximum). Values in () represent the rank of the line with respect to each loss function.

**Table A2:** Dataset 2

LINE	Posterior variance				Expected loss		
	**DTHD**	**DTMT**	**GY**	Height	KL	Energy	MALF
**Top five lines**							
177	0.39	14.82	10.25	12.88	0 (1)	—	—
202	0.38	15.22	10.53	12.52	0.002 (2)	—	—
201	0.39	15.32	10.08	12.32	0.018 (3)	—	—
64	0.38	14.90	10.28	13.05	0.034 (4)	0 (1)	0 (1)
178	0.39	15.52	10.44	13.00	0.044 (5)	—	—
35	0.39	15.22	10.45	12.69	—	0.025 (3)	0.022 (2)
38	0.38	15.03	10.35	12.75	—	0.022 (2)	0.035 (3)
211	0.37	15.48	10.57	12.81	—	0.037 (4)	0.053 (4)
213	0.39	15.25	10.15	13.05	—	0.045 (5)	0.074 (5)
**Bottom five lines**							
232	0.38	15.70	10.12	13.10	0.872 (312)	0.93 (312)	0.95 (312)
234	0.39	15.24	10.54	12.77	0.89 (313)	0.988 (315)	0.996 (314)
233	0.38	15.11	10.16	12.51	0.902 (314)	1 (316)	1 (316)
72	0.38	15.70	10.23	12.55	0.986 (315)	0.935 (313)	0.97 (313)
73	0.38	15.16	10.33	12.62	1 (316)	0.957 (314)	0.996 (315)

Five best lines and five worst lines based on posterior expected loss (PEL), under KL, Energy Score and MALF. Summaries are given for posterior variances. Values of PEL were standardized to vary from 0 (minimum) to 1 (maximum). Values in () represent the rank of the line with respect to each loss function.
