# Corrigendum to: Application of multi-trait bayesian decision theory for parental genomic selection

**DOI:** 10.1093/g3journal/jkab034

**Published:** 2021-04-22

**Authors:** Bartolo de Jesús Villar-Hernández, Sergio Pérez-Elizalde, Johannes W. R Martini, Fernando Toledo, P Perez-Rodriguez, Margaret Krause, Irma Delia García-Calvillo, Giovanny Covarrubias-Pazaran, José Crossa

In the originally published version of this manuscript, there were errors in the following co-author’s name: “Giovanny Covarrubias-Pazan” should read “Giovanny Covarrubias-Pazaran”, and “Johannes W. Martini” should read “Johannes W. R. Martini”. This has now been corrected online.

